# HLA variants and inhibitor development in hemophilia A: results from the HEMFIL study group

**DOI:** 10.1016/j.htct.2024.05.015

**Published:** 2024-09-20

**Authors:** Márcio Antônio Portugal Santana, Daniel Gonçalves Chaves, Felipe CB Souza, Suely Meireles Rezende

**Affiliations:** a Fundação de Hematologia e Hemoterapia de Minas Gerais (Hemominas), Belo Horizonte, Minas Gerais, Brazil; b Department of Internal Medicine, Faculty of Medicine, Universidade Federal de Minas Gerais, Belo Horizonte, Minas Gerais, Brazil

Hemophilia A is a hereditary bleeding disease caused by mutations in the factor VIII (FVIII) coding gene (*F8*) that lead to a reduction in the residual plasma activity of FVIII.^1^ Intravenous replacement with FVIII concentrate is the mainstay treatment of hemophilia A. However, in about 30 % of patients with severe hemophilia A, FVIII replacement triggers the development of neutralizing alloantibodies against exogenous FVIII (inhibitors),^2^ which is the main complication related to this treatment.^1^

Risk factors for the development of inhibitors can be genetic or not.^3^ The biological mechanisms involved in the immune response against FVIII are not fully understood but seem to involve a classic adaptive immune response. FVIII molecules are phagocytized by antigen-presenting cells identifying them to naïve T-helper lymphocytes through molecules of the human leukocyte antigen class II (HLA-II).^4^ As the role of HLA-II involves the presentation of peptides of FVIII to T-helper lymphocytes, it plays a crucial part in initiating the immune response against FVIII.^5^ For this reason, several studies have been carried out to investigate the role of HLA-II alleles in inhibitor development. HLA-II molecules are composed of two alpha chains and two beta chains. FVIII fragments bind to the cleft formed by the alpha 1 and beta 1 chains. The most polymorphic regions in the HLA-II genes are responsible for encoding the beta 1 chain.^6^ However, as there are 164 HLA genes in the genome, they are the most polymorphic genes in humans with more than 11,000 variants reported.^7^ This is the main barrier for investigating HLA genes as potential risk factors for inhibitor development in hemophilia A.

We investigated the association of HLA-II genotypes and the development of inhibitors in children with hemophilia A enrolled in the HEMFIL Study, a prospective cohort study aimed to identify risk factors for inhibitor development in patients with hemophilia.^8^ Patients with severe (FVIII <1 %) and moderately severe (FVIII 1–2 %) hemophilia A were enrolled before any or within five days of FVIII infusions and followed up for 75 days of treatment or until inhibitor development.^8^ For this study, in the case of siblings, only one was selected for the statistical analysis. DNA was extracted from blood samples. Polymerase chain reaction (PCR) amplification was performed to obtain specific regions of the HLA gene. Subsequently, hybridization was performed using sequence-specific oligonucleotide (SSO) probes to detect the presence of specific HLA alleles using the DNALabType SSO TM commercial test (One Lambda, Los Angeles, United States).^9^

High-definition methods were used to assess HLA-DR molecules. HLA-DQ molecules were inferred using the EPVIx software (LIB & UFPI, Teresina, Brazil).^9^ Allelic groups (low resolution) and non-specific alleles (high resolution) were analyzed to obtain a larger sample size. As the genes are codominant, the maternal and paternal molecules were analyzed concomitantly. The chi-square test was used to compare groups and a significance level of 5 % was considered significant.

Eighty-five patients were included with a median age at study enrollment of 0.8 years (interquartile range [IQR]: 0.5–1.2); 34 (40 %) developed inhibitors (INB+) at a median of 14 days of exposure (IQR: 9–21). High-risk *F8* variants (inversions of introns 1 and 22, large deletions, nonsense, frameshift mutations) were associated with inhibitor development (*p*-value = 0.007). There was no difference regarding ethnicity between the groups (*p*-value = 0.80) with 54 (63.5 %) Whites, 15 (17.6 %) Blacks, 14 (16.5 %) mixed race, one (1.2 %) native Indian and one Asian evolving with inhibitors.

The frequencies of 170 alleles (85 maternal and 85 paternal) of HLA-II DRB1 Beta, DQ Alpha-1 and DQ beta-1 chains were evaluated and no association was found of any of these alleles with inhibitor development. In the current study, only HLA DQ A1×01, which was associated with inhibitors in the study by Hay et al.^10^ (Table 2), showed a tendency for association with inhibitors (*p*-value = 0.09 - Table 1).

**Table 1:** Frequency of the HLA-DRB1, DQA1, DQB1 alleles according to inhibitor development.

Allele - n (%)	Total (*n* = 170)	INB+ (*n* = 54)	INB- (*n* = 116)	*p*-value
DRB1×01	19 (11.2)	4 (7.4)	15 (12.9)	0.31
DRB1×03	22 (12.9)	7 (13.0)	15 (12.9)	0.44
DRB1×04	14 (8.2)	6 (11.1)	8 (6.9)	0.42
DRB1×07	21 (12.4)	7 (13.0)	14 (12.1)	0.37
DRB1×08	12 (7.1)	4 (7.4)	8 (6.9)	0.56
DRB1×09	4 (2.3)	1 (1.9)	3 (2.6)	0.62
DRB1×10	2 (1.1)	1 (1.9)	1 (0.9)	0.29
DRB1×11	23 (13.5)	7 (13.0)	16 (13.8)	0.44
DRB1×12	2 (1.1)	1 (1.9)	1 (0.9)	0.29
DRB1×13	27 (15.9)	10 (18.3)	17 (10.1)	0.26
DRB1×14	4 (2.3)	2 (3.7)	2 (1.8)	0.11
DRB1×15	16 (9.3)	4 (7.4)	12 (10.3)	0.23
DRB1×16	4 (2.3)	0 (0.0)	4 (3,6)	–
DQA1×01	67 (39.4)	17 (31.5)	50 (43.1)	0.09
DQA1×02	20 (11.8)	7 (13.0)	13 (11.2)	0.36
DQA1×03	19 (11.2)	8 (14.8)	11 (9.5)	0.15
DQA1×04	14 (29.4)	4 (7.4)	10 (5.9)	0.53
DQA1×05	49 (28,6)	17 (33.3)	32 (30.3)	0.25
DQA1×06	1 (0.6)	1 (0.9)	0 (0.0)	–
DQB1×01	1 (0.6)	0 (0.0)	1 (0.85)	–
DQB1×02	40 (23.5)	14 (25.9)	26 (22.4)	0.34
DQB1×03	49 (28.8)	18 (33.3)	31 (26.7)	0.18
DQB1×04	15 (8.9)	5 (9.3)	10 (8.6)	0.54
DQB1×05	26 (15.3)	6 (11.1)	20 (17.2)	0.16
DQB1×06	39 (22.9)	11 (20.4)	28 (23.4)	0.30

INB+: inhibitor positive; INB-: inhibitor negative.

We performed a literature search and found ten studies that investigated HLA-II alleles as risk factors for inhibitor development (Table 2). The different results of these studies exemplify the variability and difficulty in establishing an association between specific HLA-II alleles and inhibitor development even in studies with large populations or historically similar ethnic groups, such as, for example, studies that enrolled only European populations (Table 2). The HLA-II DRB1×15 was the only allele associated with inhibitor development in two studies performed in ethnically different populations from Thailand and Germany (Table 2). Therefore, targeting this allele in larger studies or by grouping studies may be strategic.

**Table 2:** Studies investigating the association of HLA-II with inhibitor development in severe hemophilia A.

Author	Year	n	Study design	Population	Resolution	Inhibitor risk	Inhibitor protection
HAY et al.^9^	1997	176	retrospective cohort	British	High	DQA1×01:02	not found
OLDENBURG et al.^10^	1997	71	cross-sectional	North European	High	not found	not found
OHTA et al.^17^"?>^11^	2002	46	cross-sectional	Japanese	High	DQA1×03:01	not found
						DQ4/DR4.1	
PAVLOVA et al.^11^"?>^12^	2009	260	case-control	German	High	DRB1×15	not found
BARROS et al.^13^	2011	122	cross-sectional	Brazilian	Low	DRB1×14	not found
NATHALANG et al.^14^	2012	57	case-control	Thai	Low	DRB1×15	not found
PERGANTOU et al.^14^"?>^15^	2013	52	cross-sectional	Greek	High/Low	DRB1×01 DQB1×05:01	DRB1×11 DQB1×03
KIM et al.^15^"?>^16^	2018	100	cross-sectional	Korean	High	DRB1×13:02 DPB1×04:01	DRB1×15 DPB1×05:01
HOSSEINI et al.^13^"?>^17^	2019	101	cross-sectional	Iranian	High	not found	DRB1×01:01
McGILL et al.^4^"?>^4^	2021	997	case-control	North-American	High	DPB1×02:02	DRB1×04:07
							DRB1×11:04

In case HLA-II molecules have a role in inhibitor development in hemophilia A, it is likely that it is a weak risk factor.^10^ For a definite conclusion, studies with much larger populations are required.^5^ Furthermore, the frequency of certain haplotypes varies in different ethnic groups, and so it is not easy to compare results between populations.^10^ In highly admixed populations, such as those of Brazil and other Latin American countries, the variability is expected to be even greater. Furthermore, the technique used for HLA identification also influences comparisons between studies. High-resolution techniques such as sequence-based typing and next-generation sequencing are the most recommended due to their greater sensitivity in identifying specific alleles^9^; Although the PCR-SSO technique identifies all of them individually, it cannot directly determine haplotype information.

The main strength of this study is the well-characterized nature of the HEMFIL study and its prospective design used to detect inhibitor development. To our knowledge, this is the first study using a prospective cohort and the fourth largest to investigate this association. Despite this, the small sample size was the main barrier to study such a polymorphic gene. Another limitation is the use of a low-resolution rather than high-resolution HLA-II typing technology.

In conclusion, this study did not find any HLA-II allele associated with inhibitor development. Therefore, with larger population studies or by pooling results of studies are required to draw a definite conclusion about the role of HLA-II as a risk factor of inhibitor development in hemophilia A.

## Author contributions

MAPS collected clinical data, analyzed the data, and wrote the paper; FCBS performed HLA-II experiments, contributed to analysis and wrote the paper; DGC contributed with study design and analysis the data; SMR contributed to study design, analysis and wrote the paper. All authors revised and approved the final version of the manuscript.

## Conflicts of interest

The authors declare no competing conflict of interest.
